# Early diagnosed impacted maxillary canines and the morphology of the maxilla: a three-dimensional study

**DOI:** 10.1186/s40510-018-0220-6

**Published:** 2018-07-16

**Authors:** Giorgio Cacciatore, Laura Poletti, Chiarella Sforza

**Affiliations:** 1 Department of Biomedical Sciences for Health, Università degli Studi di Milano, Via Mangiagalli 31, 20133 Milan, Italy; 2 Private Practice in Orthodontics, Brescia, Italy

**Keywords:** Digital models, Ectopic eruption, Early diagnosis, Morphology of the maxilla

## Abstract

**Background:**

The aetiology of the canine displacement still remains controversial. Some authors implicated a deficiency in maxillary width as a local mechanical cause for impacted canines. The aim of the study was to examine whether there is a relationship between impacted maxillary canines, early diagnosed by using panoramic radiographs, and the morphology of the maxilla on 3D model casts.

**Methods:**

The displaced maxillary canines (DMC) group consisted of 24 patients (mean age, 9.1 ± 1.1 years), while the control group consisted of 25 subjects (mean age, 8.7 ± 0.9 years). Seven measurements were calculated on the digital casts of each subject: intermolar width (IMW), arch length (AL), depth of the palatal vault (PVD), available arch space (AAS), the sum of the anterior segments (SAS), the right/affected (R-Af) and left/unaffected (L-Un) available spaces.

**Results:**

Both IMW and AL in the DMC group were significantly decreased relative to the control group (*P* < 0.01), indicating that patients with displaced canines presented a shorter and narrower palate than subjects without eruption problems. Moreover, the values of the SAS and AAS were significantly decreased (*P* < 0.01) in the DMC group relative to the controls.

**Conclusions:**

The shape of the maxillary arch was narrower and shorter in the displaced maxillary canines group compared with the control group.

## Background

Displacement of maxillary canines can be defined as the ‘developmental dislocation […] often resulting in tooth impaction requiring surgical and orthodontic treatments’ [1]. The maxillary canine is second only to the mandibular third molar in its frequency of impaction, with a rate that varies from 0.2 to 2.8%. The ratio of female to male prevalence rate ranges from 1.3:1 to 3.2:1 [2].

The term ‘malposed’ or ‘displaced’ maxillary canine (DMC) is generally referred to an anomalous position of the tooth recognised at an ‘early’ stage of development [3]. From a physiological point of view, between 5 and 9 years of age, the maxillary canine tends to move palatally, with substantial movement in a buccal direction between 10 and 12 years [4]. Consequently, in the early stage of development, it is not possible to differentiate palatally displaced canines (PDC) from buccally displaced canines (BDC).

The early diagnosis (8–9 years of age) of canine displacement in relation to the surrounding structures is based primarily on radiographic examination. Methods based on panoramic radiographs [5, 6] are preferred to lateral and frontal cephalograms [4], because a panoramic radiograph is a primary routine investigation for many patients. In particular, diagnosis of maxillary canine impaction is possible at 8 years of age by using the following geometric measurements on panoramic radiographs: sector locations of impacted maxillary canines, angulations formed by the long axis of the impacted canine with the midline, and the distance of the cuspal tip of the impacted canine and the unaffected antimere from the occlusal plane [6].

The aetiology of the canine displacement still remains controversial. Crowding may play a role as an environmental cause of impaction, although arch length deficiency is associated primarily with buccal canine impaction [7]. Two major theories have been delineated to explain the occurrence of PDC, i.e. the ‘guidance’ theory [8] and the ‘genetic’ theory [2]. The ‘sequential hypothesis’ provides a sequence, in which the two most commonly accepted theories might act at different stages during the development of the maxillary canine and the surrounding structures [9]. Mesial intraosseous displacement of the maxillary first premolar is also significantly associated with the displacement of the permanent canine in the intermediate mixed dentition [10].

A different aetiology was discussed by McConnell et al. [11], who implicated a deficiency in maxillary width as a local mechanical cause for palatally impacted canines. A literature review about the relationship between displaced/impacted maxillary canines and the intermolar width was conducted before starting the current study [7, 11–17]. The results of this review are presented in Table 1. Some authors stated that an association between PDC and transverse discrepancies could be present [14, 15], but most of the authors did not find differences in intermolar width of PDC and control groups [7, 11, 13, 16, 17]. In contrast, Al-Nimri and Gharaibeh [12] found that the width at maxillary first molar was greater in patients with PDC. Only one study evaluated patients aged under 10 years [14], whereas the patient ages of the remaining investigations ranged between 10 and 42 years [7, 11–13, 15–17]. Moreover, different methods of measurement were used: cone-beam computed tomography [16, 17] and dental casts [7, 11–15].

**Table 1:** Literature review about the relationship between displaced/impacted maxillary canines and the intermolar width

Authors	Participants	Controls	Mean age of participants (years)	Mean age of controls (years)	Methods of measurement	Association between IMW and PDC
Hong et al. [17]	PDC	Not PDC	18.2	18.1	CBCT	No association
Yan et al. [16]	PDC-BDC	Not PDC-BDC	21.0	Matched with participants	CBCT	No association
Kim et al. [15]	PDC	BDC	12.8	12.1	Dental casts	Decreased IMW associated with PDC
Schindel and Duffy [14]	PXB	Not PXB	9.5	9.9	Dental casts	Decreased IMW associated with PDC
Saiar et al. [13]	PDC	Not PDC	12.2	12.2	Dental casts	No association
Al-Nimri and Gharaibeh [12]	PDC	Not PDC	17.7	Matched with participants	Dental casts	Increased IMW associated with PDC
Langberg and Peck [7]	PDC	Not PDC	13.6	Matched with participants	Dental casts	No association
McConnell et al. [11]	PDC or BDC	Not PDC-BDC	–	–	Dental casts	No association

*PDC* palatally displaced or impacted canines, *BDC* bucally displaced or impacted canines, *PXB* posterior crossbite, *IMW* width at maxillary first molar, *CBCT* TC cone beam

Overall, the results of the previous investigations are still unclear, and clinical data in children in their first decade of life are still scanty. No previous studies evaluated the relationship between displaced maxillary canines and the morphology of the maxilla on digital casts. The aim of this study was to examine whether there is a relationship between impacted maxillary canines, early diagnosed by using panoramic radiographs and the morphology of the maxilla on 3D model casts. If an association between some characteristics of the palate and displaced maxillary canines was demonstrated early, the shape of the palate could be changed with an orthodontic treatment.

## Methods

### Study design

Subjects aged 7 to 11 years who received a periodical orthodontic evaluation at a single private practice of one of the authors (GC) between 2012 and 2015 were considered for inclusion. Early prediction of maxillary canine impaction was made by using geometric measurements on panoramic radiographs. The measurements included the position (sector) and angulation of the tooth and the distance from the occlusal plane (adapted from Ericson and Kurol [5]).

**Fig. 1:**
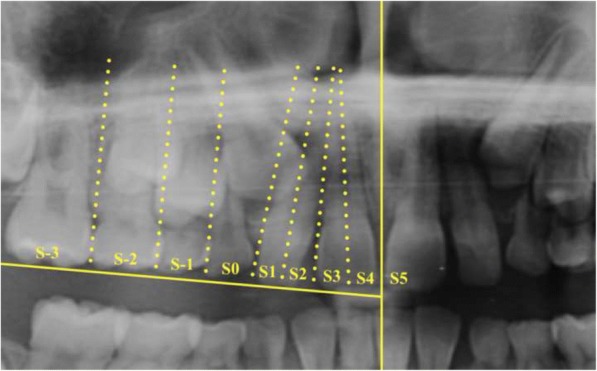
Diagrammatic representation of the most common sectors of the impacted canine [5]

**Fig. 2:**
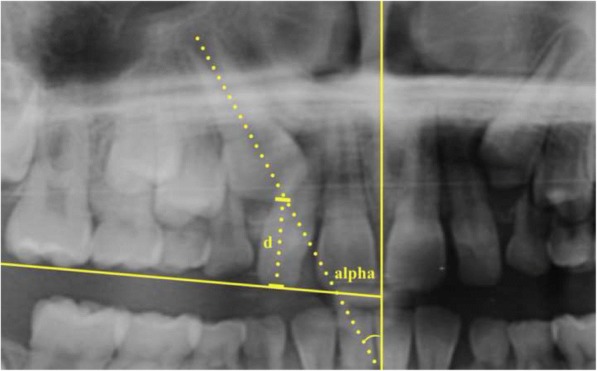
Diagrammatic representation of the measurement of the angulation and the distance from the occlusal plane [5]

The classification of sectors depended on the location of the tip of the impacted canine relative to the surrounding teeth (Fig. 1). The angle α was made by the long axis of the impacted maxillary canine with the midline, defined by the following landmarks on the radiograph: intermaxillary suture, anterior nasal spine, nasal septum and internasal suture. The distance from the occlusal plane (*d*) was measured on the perpendicular line drawn from the incisal tip of the impacted canine to the occlusal plane. The occlusal plane was determined by drawing a horizontal line passing through the incisal edge of the central permanent incisor and the occlusal plane of the first permanent molar on the given side (Fig. 2).

Inclusion criteria of early diagnosed impacted maxillary canines, according to Sajnani et al. [6], were as follows: (1) sector of impacted maxillary canine different from S0, (2) angulation larger than 30.0°, and (3) distance from the occlusal plane larger than 20.0 mm. Patients with bilateral impaction were included only if the position of impaction was the same on both sides. All radiographs were examined in a darkened room by using an illuminated x-ray viewing box. The panoramic radiographs were traced with 0.003-in matte acetate tracing paper and a 0.5-mm HB fine lead pencil.

In addition, the exclusion criteria were as follows: (1) previous orthodontic treatment, (2) patients with definitive obstructions (e.g. odontoma or supernumerary teeth), (3) patients with a systemic disease, (4) patients with craniofacial anomalies (e.g. cleft lip or palate) and (5) patients with several impacted teeth or congenitally missing teeth.

### Digital cast analysis

The upper dental cast of all subjects was obtained from A-Silicone impressions (Elite HD+, Zhermack SpA, Badia Polesine, Italy). The dental cast was scanned by a three-dimensional scanner (D100, Imetric 3D, Courgenay, Switzerland) and analysed by the VAM software (Vectra 3D, Canfield Scientific, Fairfield, NJ). The three-dimensional scanner projects a pattern of structured light on the object and looks at the deformation of the pattern on the object. A camera looks at the shape of the pattern and calculates the distance of every point in the field of view. Data are collected in relation to an internal coordinate system; thus, the scanner creates a three-dimensional image.

**Fig. 3:**
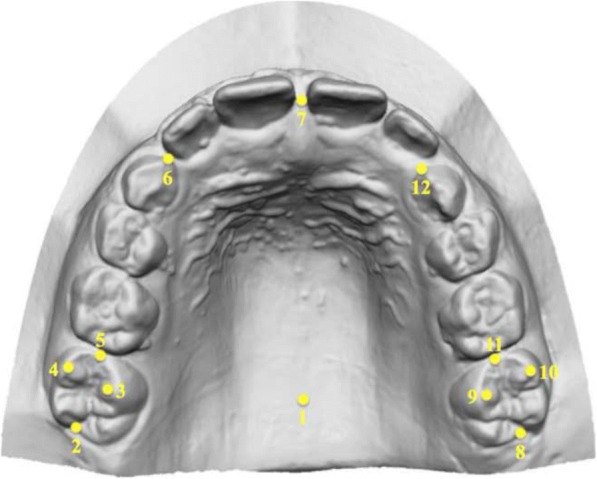
Example of 12 standardised dental landmarks: 1: the deepest point of the palatal vault; 7: contact point between the central incisors; 2, 8: distal ends of the right and left first molars; 3, 9: mesio-palatal cusp tips of the first molars; 4, 10: mesio-buccal cusp tips of the first molars; 5, 11: mesial ends of the right and left first molars; 6, 12: mesial contact points of the right and left primary canines

**Fig. 4:**
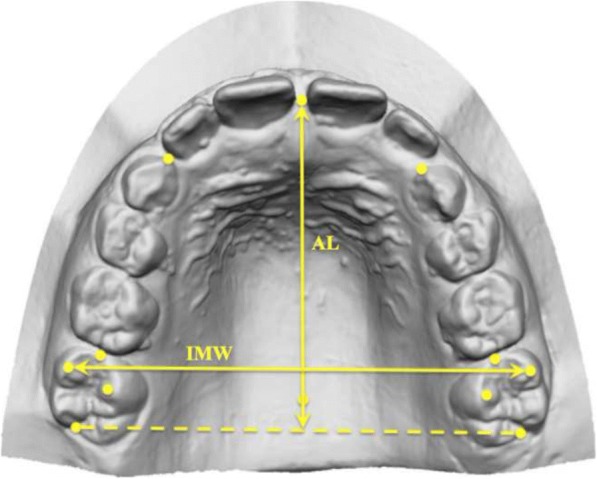
Representation of the measurements of the intermolar width (IMW) and arch length (AL)

**Fig. 5:**
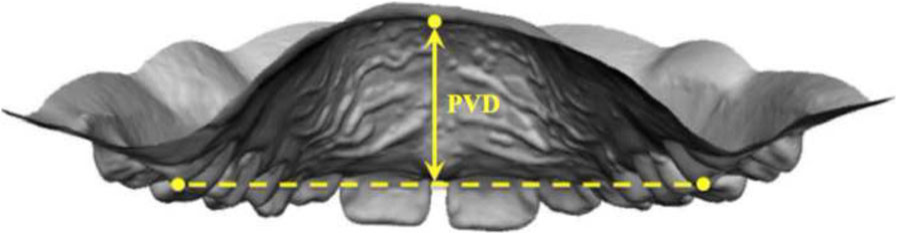
Representation of the measurement of the depth of the palatal vault (PVD)

**Fig. 6:**
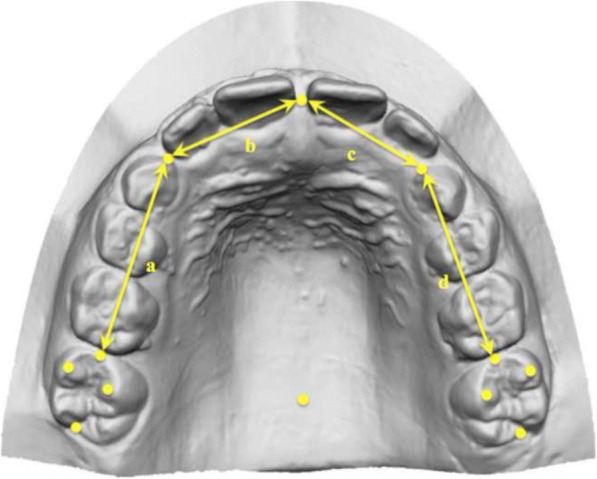
Representation of the space measurements. Sum of the anterior segments (SAS) = b + c; available arch space (AAS) = a + b + c + d; right/affected (R-Af) available space = a + b; left/unaffected (L-Un) available space = c + d

On all casts, a set of 12 standardised dental landmarks was identified (Fig. 3), as previously described by Kim et al. [15]. The three-dimensional (*x*, *y*, *z*) coordinates of the landmarks were obtained, and a customised Excel spread-sheet (Microsoft Excel, Microsoft, Redmond, WA) was used for all the subsequent 3D calculations:

- Intermolar width (IMW) was defined as the distance between the mesio-buccal cusp tips of the first molars (Fig. 4);
- Arch length (AL) was defined as the distance from the contact point between the central incisors to the line that links the distal ends of the right and left first molars. If the antero-posterior position of the left and right maxillary central incisors differed for reasons including crowding, the values on the right and left sides were measured, and the average value was used;
- Depth of the palatal vault (PVD) was defined as the vertical distance from the deepest point of the palatal vault to the contact line between the mesio-palatal cusp tips of the right and left first molars (Fig. 5);
- The upper arch was divided into four segments: two segments from the mesial ends of the right and left first molars to the mesial contact points of the right and left primary canines, two segments from the primary canines to the contact point between the central incisors. Available arch space (AAS) was estimated with the sum of these four segments, while the sum of the widths of the four maxillary incisors was estimated with the sum of the two anterior segments (SAS, Fig. 6);
- Moreover, in the control group, the right (R) and left (L) available space was estimated respectively with the sum of the two right and left side segments. In the DMC group, all the patients with unilateral impaction were considered to be affected (Af) on the right side, while the left side was considered to be the unaffected side (Un).

The digitizer resolution was 0.013 cm/cm of range and its accuracy 0.025 cm. Digitization of landmarks was performed by a single operator (GC).

### Error of the method and power of the study

The level of significance was fixed at 0.01 for all statistical tests. Seven dental casts were randomly selected from both groups and redigitized by the same operator. The variables were recalculated to determine the method error with the intraclass correlation coefficient (ICC). The ICCs ranged from 0.87 to 0.99 for all examined variables.

The power of the study for the unpaired *t* test was assessed on the basis of the sample size of the DMC and control samples, an alpha level of 0.01, with a mean difference for the clinically relevant variable (IMW) of 2.54 mm and with a standard deviation of 2.48 mm. The calculated power was 0.86 (SigmaStat version 3.5, Systat Software, Point Richmond, CA).

### Statistical analysis

The chi-square test and two-way factorial ANOVA for independent samples were calculated to compare respectively the female to male ratio and the ages of the samples. No statistically significant differences were found both in the female to male ratio (chi-square = 0.22; *P* = 0.639) and the ages (F = 1.21; *P* = 0.277) of the two groups. The normal distribution and homoscedascity of the samples were checked before starting inferential analysis, by using the Shapiro-Wilk test and Levene’s test, respectively.

Preliminary *t* tests between patients with bilateral and unilateral displacement were executed. As no statistically significant differences were recorded between the two groups in all variables, patients with bilateral and unilateral displacement were considered parts of the same group (DMC group). Therefore, differences between the DMC group and the control group were calculated directly by means of Student’s *t* test for independent samples. On the contrary, the differences between the right/affected and left/unaffected sides were evaluated by paired samples *t* tests (Microsoft Excel, Microsoft, Redmond, WA). The effect size (ES) coefficient was also calculated [18]. For Cohen’s *d*, an effect size of 0.2 to 0.3 could be considered a ‘small’ effect; around 0.5, a ‘medium’ effect; and 0.8 to infinity, a ‘large’ effect.

## Results

Subjects were divided into two groups: the DMC group and the control group. The DMC group consisted of 24 patients, female to male ratio 2:1, with a mean age of 9.1 ± 1.1 years, while the control group consisted of 25 subjects, female to male ratio 14:11, with a mean age of 8.7 ± 0.9 years. Fourteen patients had bilateral maxillary canine impactions. Most of the patients (19 of 24 patients, 32 of 38 maxillary canines in the DMC group, 22 of 25 subjects in the control group) were under 10 years.

The means, standard deviations and results of Student’s *t* test for independent samples between the two groups are shown in Table 2, while the values of the comparisons between the right/affected and the left/unaffected sides of both groups are given in Table 3.

**Table 2:** Results of statistical comparisons between the groups

Measurements (mm)	DMC		Controls				Effect size	
	Mean	SD	Mean	SD	*P* value	Significance	*d* value	ES
IMW	47.21	2.48	49.75	2.19	0.00042	**	1.06119	L
AL	36.46	1.92	38.16	2.13	0.00510	**	0.82173	L
PVD	14.73	1.11	14.67	1.49	0.87840	NS	–	–
SAS	29.15	2.08	31.97	2.32	0.00005	**	1.25026	L
AAS	73.82	2.73	77.88	3.23	0.00002	**	1.32721	L
R-Af	36.75	1.78	38.82	1.78	0.00018	**	1.13612	L
L-Un	37.07	1.23	39.06	1.57	0.00001	**	1.37753	L

*DMC* displaced maxillary canines, *IMW* intermolar width, *AL* arch length, *PVD* depth of the palatal vault, *SAS* sum of the anterior segments, *AAS* available arch space, *R-Af* right/affected, *L-Un* left/unaffected available space, *SD* standard deviation, ** statistically significant (*P* < 0.01), *NS* not significant, *ES* effect size, *L* large

**Table 3:** Results of statistical comparisons between the right/affected and left/unaffected sides

Groups	R-Af (mm)		L-Un (mm)				Effect size	
	Mean	SD	Mean	SD	*P* value	Significance	*d* value	ES
DMC	36.75	1.78	37.07	1.23	0.26364	NS	–	–
Controls	38.82	1.78	39.06	1.57	0.20924	NS	–	–

*DMC* displaced maxillary canines, *R-Af* right/affected, *L-Un* left/unaffected available space, *SD* standard deviation, ** statistically significant (*P* < 0.01), *NS* not significant, *ES* effect size

Regarding the comparison between the DMC and control groups, both IMW and AL in the DMC group were significantly decreased relative to the control group (*P* < 0.01), indicating that patients with displaced canines presented a narrower and shorter palate than subjects without eruption problems. The greater difference between both groups was registered in the IMW (2.5 mm), while the difference in the AL was 1.7 mm.

No statistically significant differences between the two groups were found in the PVD.

Moreover, the values of the SAS and AAS used to determine eruption space were significantly decreased (*P* < 0.01) in the DMC group relative to the controls. These findings are consistent with those found for AL and IMW. The right/affected and left/unaffected sides were shorter in the DMC group as well (*P* < 0.01), although there were no statistically significant differences between the two sides (right/affected, left/unaffected) in both groups.

All statistically significant variables (IMW, AL, SAS, AAS, R-Af, L-Un) were also characterised by a significant large effect size.

## Discussion

The aim of the present study was to examine whether there is a relationship between displaced maxillary canines and the morphology of the maxilla in the early mixed dentition, on 3D model casts. Though many articles have been previously published on this topic, their results are controversial and, sometimes, contradictory [7, 11–17]. No previous studies evaluated the relationship between impacted maxillary canines, early diagnosed by using panoramic radiographs, and the morphology of the maxilla, on digital models.

Finding an association between DMC and the morphology of the maxilla at an early stage is extremely important, as orthodontists could change the shape of the palate with their treatments [19]. Complications described for early treatment [20] were fewer than those described for the surgical exposure of the crown of the canine followed by orthodontic traction of the impacted tooth [21]. Moreover, with their advantages in terms of cost, time and space required, digital models could be considered the new gold standard in current practice [22]. Even if digital models were obtained by scanning plaster casts, applicability to digital impressions with intraoral scanners seems to be feasible.

As ages of most of the patients (19 of 24 patients) were under 10 years, most of the DMC (32 of 38 maxillary canines) could be considered as PDC. Buccal movement of maxillary canines usually occurs between the ages of 10 and 12 years [4]. Meanwhile, female to male ratios in the DMC and control groups are consistent with those found in other studies and in the normal population, respectively [2].

The most important finding of this study was that both intermolar width (IMW) and arch length (AL) were significantly decreased (*P* < 0.01) in the DMC group relative to the controls, indicating that patients with maxillary canines which could have some problems during the eruption process presented a narrower and shorter palate compared with subjects without any eruption problems.

The reduction of the IMW was consistent with the results found by Schindel and Duffy [14] and Kim et al. [15], but in contrast with those by other authors that did not find any statistically significant differences between patients with PDC and the controls [7, 11, 13, 16, 17]; Al-Nimri and Gharaibeh [12] even stated that the transverse arch dimension was significantly wider in the impaction group than in the comparison group. These discrepancies can be explained by the significant heterogeneity (age, gender, ethnicity, inclusion criteria, methods of measurement) found in other papers.

The reduction of the AL was in contrast with the two articles that investigated this measurement [11, 15], but it was in agreement with the findings by Baccetti et al. [23] and Mucedero et al. [10]. Baccetti et al. [23] showed that a significant mesial movement of the upper first molars (about 2.5 mm) occurred in subjects with untreated PDC, while Mucedero et al. [10] asserted the mesial intraosseous displacement of the maxillary first premolar is significantly associated with the displacement of the permanent canine in the intermediate mixed dentition.

The present study compared the depth of the palatal vault (PVD) between patients with DMC and a control group of patients without eruption problems first. No statistically significant differences were found in the PVD between the two groups. Kim et al. [15] also evaluated the PVD, but they compared a PDC group with a BDC group. A deeper palatal vault was observed in patients with PDC relative to those with BDC. No other authors among those studying dental arches in patients with impacted canines investigated this measurement [7, 11–14, 16, 17].

Consistently with the reduction of IMW and AL, the sum of the anterior segments (SAS) and the available arch space (AAS) were also significantly decreased in the DMC group compared with the control group (*P* < 0.01). If the upper dental arch was considered round, the IMW could be estimated as the diameter of the circumference and the AAS as half of the arch perimeter. The SAS is part of this perimeter. The interrelation among IMW, AL, SAS and AAS can explain the uniformity of these findings. On the contrary, Kim et al. [15] did not find any statistically significant differences in the eruption space between the palatally and buccally impacted canine groups.

Likewise, the right/affected and left/unaffected sides were shorter in patients with DMC relative to the controls (*P* < 0.01), although there were no statistically significant differences between the two sides (right/affected, left/unaffected) in both groups. A possible explanation is that the number of patients with bilateral displaced canines was greater than that with unilateral impaction. This result disagrees with the work by Talinada et al. [24]. They evaluated the alveolar arch perimeter discrepancy in unilateral palatally impacted canines, finding that there was a significant decrease in the arch perimeter on the impacted side.

The statistically significant differences between the DMC group and the controls were also clinical significant, as all variables (IMW, AL, SAS, AAS, R-Af, L-Un) were characterised by a large effect size.

**Fig. 7:**
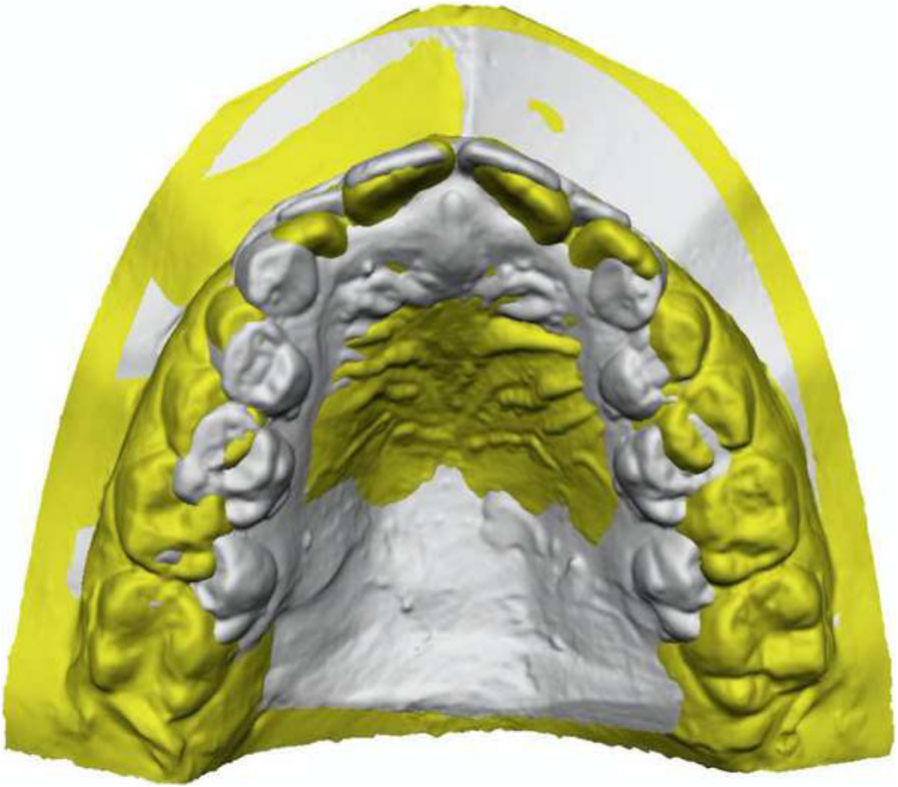
Comparison between a patient with DMC (grey) and a control subject (yellow): occlusal view

**Fig. 8:**
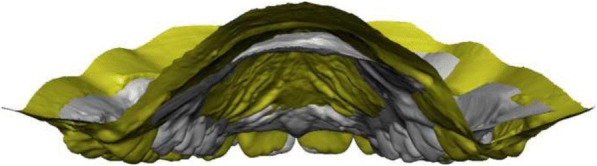
Comparison between a patient with DMC (grey) and a control subject (yellow): posterior view

An exemplifying comparison between a patient with DMC and a subject without eruption problems is illustrated in Figs. 7 and 8. Palatal rugae [25, 26] and the contact point between the central incisors were used for the superimposition of the upper digital casts. It is evident how the width and length of the DMC patients were reduced, whereas no differences could be observed in the symmetry of the dental models.

The findings of the current study have a direct clinic application, because they corroborate some interceptive procedures aimed to increase maxillary arch width and length, so the arch perimeter, as preventive protocols proposed for displaced maxillary canines. Currently, rapid maxillary expander (RME) and cervical-pull headgear (HG) are orthodontic treatments proposed for displaced maxillary canines also validated by a systematic review [19]. As the systematic reviews reach the highest level of scientific evidence, the strength of the consistency between the findings of the reduction of IMW and AL and the effectiveness of protocols that increase maxillary arch width and length is raised.

As anticipated, some limitations occurred in the current study. First of all, no distinction between female and male patients was made, so it cannot be deduced if differences in the morphology of the maxilla relied on gender exist. Additionally, the DMC and control samples were composed by only European patients, who, according to literature, exhibit more prevalence of canine impaction than African or Asian subjects [2]. So, the extension of the present results to other populations should be verified.

In addition, the most significant limitation of the work concerned the group of DMC that included unilateral as well as bilateral maxillary canine impactions. The influence of the type of impaction on the shape of the palate remains unclear. However, the reduced sample size did not allow further analyses.

More research is needed to overcome these limitations. A comprehensive study which considers the different prevalence of the maxillary canine impaction in patients of different genders or ethnic origins, and evaluate the aetiology of unilateral/bilateral or palatal/buccal impactions, should be encouraged. Similarly, the association between the upper canine displacement and other dental anomalies (peg-shaped lateral incisors, missing teeth, etc.), or the assessment of maxillary volume, could help clinicians to better understand and face this phenomenon. Surely, the possibility to collect digital casts makes the realisation of a multicentre study possible and the extension of the sample easier.

## Conclusions

The aim of this study was to examine whether there is a relationship between impacted maxillary canines, early diagnosed by using panoramic radiographs and the morphology of the maxilla on 3D model casts.

- Both IMW and AL in the DMC group were significantly decreased relative to the control group, indicating that patients with displaced canines presented a narrower and shorter palate than subjects without eruption problems.
- Moreover, the values of the SAS and AAS used to determine eruption space were significantly decreased in the DMC group relative to the controls. These findings are consistent with those found for AL and IMW.
- Further research is needed to overcome limitations of the current study. A comprehensive study with a larger sample size, which considers the different prevalence of the maxillary canine impaction in patients of different genders or ethnic origins, and evaluate the aetiology of unilateral/bilateral or palatal/buccal impactions, should be encouraged.

## Abbreviations

AAS: Available arch space
AL: Arch length
BDC: Buccally displaced canines
CBCT: TC cone beam
DMC: Displaced maxillary canines
ES: Effect size
HG: Cervical-pull headgear
ICC: Intraclass correlation coefficient
IMW: Intermolar width
L-Un: Left/unaffected available space
PDC: Palatally displaced canines
PVD: Depth of the palatal vault
PXB: Posterior crossbite
R-Af: The right/affected available space
RME: Rapid maxillary expander
SAS: The sum of the anterior segments
